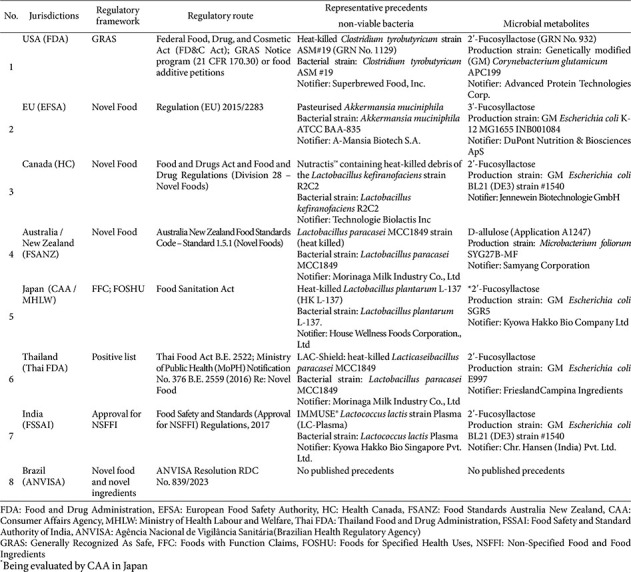# Key Safety Criteria for Novel Microbial Food Ingredients: A Comparative Analysis of International Regulatory Frameworks

**DOI:** 10.4014/jmb.2605.05032

**Published:** 2026-07-20

**Authors:** Yejin Choi, Ji-Seon Hwang, Hyunok Doo, Jinok Kwak, Juyoun Kang, Haram Kim, Yeongjae Chae, Suyoung Lee, Sheena Kim, Ju-Hoon Lee, Hyeun Bum Kim

**Affiliations:** 1 Department of Animal Biotechnology, Dankook University, Cheonan 31116, Republic of Korea; 2 Department of Agricultural Biotechnology, Seoul National University, Seoul 08826, Republic of Korea; 3 Department of Food and Animal Biotechnology, Seoul National University, Seoul 08826, Republic of Korea; 4 Center for Food and Bioconvergence, Seoul National University, Seoul 08826, Republic of Korea; 5 Research Institute of Agriculture and Life Sciences, Seoul National University, Seoul 08826, Republic of Korea

**Keywords:** Food safety, Novel microorganisms, Non-viable bacteria, Metabolites

## Abstract

In the context of the research on food ingredients, “novel microorganisms (NMs)” refers to microorganisms without a documented history of safe consumption. The utilization of these novel microorganism-derived ingredients is expanding rapidly within the food and nutrition sectors, and such ingredients are broadly classified into three categories, live microorganisms, non-viable bacteria, and purified metabolites, each characterized by distinct risk profiles and unique regulatory requirements. Despite their growing prevalence, a globally harmonized protocol for safety assessment is currently lacking, leading individual nations to implement disparate safety frameworks within their respective regulatory systems. Consequently, this review aimed to provide a scientific foundation for the standardization of safety criteria by performing a comparative analysis of established guidelines from the European Union (EFSA), United States (FDA), Canada (Health Canada), Brazil (ANVISA), Thailand (Thai FDA), India (FSSAI), Japan (FSCJ), and Australia/New Zealand (FSANZ). Our analysis identified several universal safety parameters for live NMs, including hemolytic activity, antimicrobial resistance (AMR), toxigenic potential, the production of antimicrobial substances, metabolic profiling, and toxicological assessment. While toxicological evaluation, the production of antimicrobial substances, and the absence of live NMs were the consensus criteria for the safety evaluation of non-viable bacteria and purified microbial metabolites, an allergenicity evaluation is additionally required for purified microbial metabolites. Based on our analysis, this review proposed safety standards for novel microbial food ingredients including live microorganisms, non-viable bacteria, and purified metabolites by synthesizing these diverse regulatory landscapes.

## Introduction

Microorganism-derived food ingredients have gained global interest as their applications expand within functional foods, next-generation probiotics (NGPs), and health-targeted nutritional products [1, 2]. In alignment with these trends, safety assessment parameters for live microorganisms used as food ingredients are being more rigorously enforced, encompassing both those with a documented history of safe use and novel taxa.

For example, *Lactobacillus* and *Bifidobacterium* are widely recognized as traditional probiotics with a long history of safe use [3, 4]. However, even strains from these established genera require a comprehensive safety assessment before being used as food ingredients because regulatory agencies such as the European Food Safety Authority (EFSA) and the U.S. Food and Drug Administration (FDA) require safety assessments of those probiotics at the strain level, recognizing that even minor genomic differences can significantly affect functionality, safety, and host interactions [5-8].

In the context of the research on food ingredients, “novel microorganisms (NMs)” refers to a microorganism without a documented history of safe consumption. For the NMs, more stringent safety assessments are required before their application as food ingredients. Good examples of these cases are *Akkermansia muciniphila* and *Faecalibacterium prausnitzii*, which have potential for anti-inflammatory and metabolic health benefits. However, these bacteria have a limited history of use as food and lack evidence for safe consumption. Therefore, regulatory agencies including EFSA and FDA require more stringent safety assessments before their application as food ingredients [1]. The regulatory agencies are facing more such cases because the advancement of high-throughput sequencing and multi-omics technologies has significantly enhanced the capacity to identify microbial taxa associated with specific physiological and health-related traits distinct from those traditionally used as probiotics [9-13].

In addition to probiotic uses, the NMs are being actively explored in a wide range of food applications in the form of non-viable bacteria and metabolites. These include single-cell protein production as an alternative protein source, microbial synthesis of nutrients and functional ingredients such as vitamins, amino acids, and natural pigments, and the use of microbial byproducts like short-chain fatty acids and cell wall components in postbiotic development [14-17]. These emerging applications further emphasize the need for consistent and rigorous microbial safety evaluations.

Despite common objectives, pronounced variations exist in the empirical data requirements and the depth of risk assessment protocols for novel microbial food ingredients across jurisdictions. Through a synthesis of these divergent regulatory landscapes, this review established a scientific foundation for the harmonization of safety standards for novel microbial food ingredients, encompassing live microorganisms, non-viable bacteria, and metabolites.

### Definition and Classification of Microorganisms as Novel Food

In the context of novel foods, “microorganism-derived ingredients consisting of, isolated from, or produced with microorganisms lacking a history of safe human consumption” are classified as novel foods. The criteria for determining a history of safe use vary across jurisdictions. For instance, the EFSA uses May 15^th^ 1997, as the reference date; any food ingredient not consumed significantly within the EU before this date qualifies as novel. Conversely, the FDA utilizes January 1^st^ 1958, as the threshold for evaluating a history of safe use under the Generally Recognized as Safe (GRAS) framework [18, 19]. Beyond chronological thresholds, the classification of novel foods often extends to international consumption history, which can serve as supportive evidence for safety, particularly when the domestic history of use is limited.

While no universally harmonized definition currently exists for novel microorganism-derived food ingredients, various regulatory entities utilize distinct classification schemes. For example, the EFSA defines novel foods consisting of viable, non-genetically modified microorganisms (non-GMMs) as “active agents.” Meanwhile, novel foods derived from non-GMMs in which inactivated cells or their genetic material remain detectable are defined as “biomass.” [19].

**Fig. 1:**
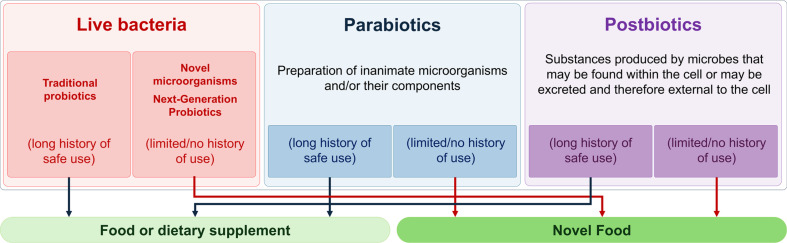
Definition and Classification of Microorganisms as Novel Food. The framework incorporates live bacteria, parabiotics, and postbiotics proposed by Wortelboer *et al*. (2022) [20].

Previously, Wortelboer *et al*. proposed a classification system for microbial products based on viability, biological composition, history of use, and intended application. This system encompasses three categories: (i) live bacteria (viable microorganisms), including NGPs lacking a history of safe use and genetically modified microorganisms (GMMs); (ii) parabiotics, consisting of non-viable cells; and (iii) postbiotics, comprising non-viable bacterial products or metabolites (Fig. 1) [20].

Nevertheless, the classification approaches proposed by the International Scientific Association for Probiotics and Prebiotics (ISAPP) have been widely adopted by international regulatory authorities and in peer-reviewed literature. The ISAPP provides consensus definitions for probiotics and postbiotics while establishing a clear conceptual distinction for microbial metabolites (Table 1). The ISAPP defines probiotics as “live microorganisms that, when administered in adequate amounts, confer a health benefit on the host” and postbiotics as a “preparation of inanimate microorganisms and/or their components that confers a health benefit on the host” [21]. Crucially, the ISAPP clarifies that purified microbial metabolites, in the absence of inanimate microbial biomass or components, do not meet the definition of postbiotics and should instead be categorized separately as microbial metabolites [22].

Based on our analysis, we propose systematic definitions and classification criteria for emerging categories of novel microorganism-derived food ingredients. Building on the regulatory and scientific perspectives outlined above, this review categorizes these ingredients into three distinct groups: (i) “live novel microorganisms (NMs),” which refer to live bacteria without a documented history of safe consumption based on the chronological thresholds of either EFSA or the FDA; (ii) “non-viable bacteria”; and (iii) "purified microbial metabolites.

### Global Regulatory Frameworks for the Safety Assessment of Novel Microorganism-Derived Food Ingredients Compared in This Review

Regulatory frameworks for the safety assessment of novel microorganism-derived food ingredients vary across jurisdictions in their definitions of novelty, oversight approaches, and data requirements. Given the absence of harmonized international standards, a cross-country comparison was conducted to identify shared regulatory principles and major points of divergence.

The jurisdictions included in this comparison were selected based on the following criteria: (i) the presence of an internationally recognized regulatory system with influence on safety assessment practices; (ii) the existence of established regulatory frameworks applicable to microorganism-derived food ingredients with documented evaluation cases; (iii) the availability of publicly accessible regulatory documents and review materials, and (iv) the representation of diverse geographical regions to provide a comprehensive view of global regulatory trends. Based on these criteria, the United States (Food and Drug Administration, FDA), the European Union (European Food Safety Authority, EFSA), Canada (Health Canada, HC), Australia/New Zealand (Food Standards Australia New Zealand, FSANZ), Japan (The Food Safety Commission of Japan, FSCJ & Consumer Affairs Agency, CAA), Thailand (Thailand Food and Drug Administration, Thai FDA), India (Food Safety and Standards Authority of India, FSSAI), and Brazil (Agência Nacional de Vigilância Sanitária, ANVISA) were selected for analysis (Table 2). This comparison aimed to identify both the commonalities and divergences among these national regulatory systems and to summarize major considerations, such as specific safety evaluation criteria, relevant to the assessment of novel microbial ingredients.

### Safety Assessment Criteria for live NMs as Novel Food Ingredients

Regulatory agencies within the evaluated jurisdictions apply distinct safety evaluation criteria to novel microorganism-derived food ingredients, leading to varying safety data requirements [19]. Even though all available evidence regarding the pathogenic potential of the microorganism or its closely related species should be considered to ensure the strain does not pose a safety risk, the safety evaluation of live NMs as food ingredients generally relies on a common set of core assessment criteria designed to address potential health risks [23, 24].

As summarized in Table 3, required safety considerations in the case of live NMs include: (1) antimicrobial resistance; (2) antimicrobial production; (3) virulence and pathogenicity factors; (4) metabolic safety; and (5) toxicological evaluation [25]. Likewise, the safety of genetically modified microorganisms (GMMs) is assessed under dedicated regulations for genetically modified organisms (GMOs). For the safety assessment, jurisdictions including the EU (EFSA) and the Republic of Korea (MFDS) [26-30] commonly require: (1) characterization of the host and donor strains; (2) molecular characterization of the genetic modification, established by comparison with the non-modified parental strain; (3) toxicological and (4) allergenicity assessment of the newly expressed protein(s); and (5) nutritional assessment relative to a conventional counterpart. Nevertheless, GMMs lie outside the scope of this review and will not be discussed further.

### Assessment of Antimicrobial Resistance

The evaluation of antibiotic resistance of bacteria is considered one of the most fundamental and critical components in food safety evaluation [31, 32]. Therefore, all jurisdictions compared in this review require the assessment of antimicrobial resistance for NMs as food ingredients (Table 3). Antimicrobial resistance is assessed through *in vitro* methods, such as minimum inhibitory concentration (MIC) testing, with results compared against established cut-off values provided by authorities such as the EFSA [33]. More recently, this phenotypic assessment has been complemented by whole-genome sequencing (WGS)-based approaches, enabling the identification of antibiotic resistance genes and the assessment of their genetic context [34-36]. In particular, determining whether resistance genes are associated with mobile genetic elements is essential for evaluating the potential for horizontal gene transfer (HGT), thereby minimizing the risk of dissemination of resistance traits to other microorganisms [37]. Intrinsic resistance, which is a natural characteristic of the microbial species, is generally considered acceptable. In contrast, acquired resistance requires additional evaluation to determine its origin. Resistance arising from chromosomal mutations in native genes may be acceptable, whereas resistance associated with acquired genes, particularly those linked to mobile genetic elements, is considered a safety concern and may preclude the use of the microorganism [38].

### Evaluation of Antimicrobial Production

Most jurisdictions compared in this review require the assessment of antimicrobial production by NMs intended as food ingredients (Table 3). This assessment is conducted to determine whether the candidate microorganism, or its closely related species, produces substances that may interfere with clinically important antimicrobials [19, 39]. Specifically, the evaluation focuses on the potential production of compounds classified as Critically Important Antimicrobials (CIAs) or Highly Important Antimicrobials (HIAs) as defined by the World Health Organization (WHO) [39, 40]. These compounds encompass conventional antibiotics as well as other antimicrobial substances, such as bacteriocins. According to EFSA guidelines, antimicrobial activity is evaluated by testing the inhibitory effects of culture supernatants against reference strains [41]. If growth inhibition is observed, the responsible compound must be identified. If the inhibitory substance is identified as an antimicrobial agent listed on the CIA or HIA registry, its use is unacceptable unless it is demonstrated to be absent from the final product. Conversely, naturally produced antimicrobial metabolites, such as organic acids or bacteriocins, are generally acceptable, provided that all relevant safety concerns are addressed.

### Virulence Factor Analysis and Pathogenic Potential

The evaluation of virulence and pathogenicity factors is essential to assess the potential of the microorganism to exert adverse effects on the host [30]. Consequently, all jurisdictions compared in this review require the characterization of pathogenic potential for NMs intended as food ingredients (Table 3). This evaluation relies on a combination of genomic and phenotypic analyses. For instance, WGS is widely employed to screen for virulence-associated determinants, such as those encoding toxin production, adhesion, and invasion, by comparing sequences with established virulence factor databases [42].

Phenotypic characterization of NMs includes the assessment of cytotoxicity using assays such as lactate dehydrogenase (LDH) release, which reflects host cell membrane damage. The LDH assay serves as a critical tool in the safety assessment of NMs intended for food applications, specifically to evaluate the potential cytotoxic effects of candidate microbial strains or their secreted metabolites on human intestinal epithelial cells. Upon oral consumption, these microorganisms establish direct contact with the intestinal epithelium, therefore demonstrating the absence of epithelial damage is essential to establish their suitability for human consumption. Cytotoxicity levels comparable to the negative control or within an acceptable baseline threshold are considered safe, whereas intermediate levels necessitate further evaluation integrated with genomic data [43].

### Assessment of Hemolytic Activity

Hemolytic activity indicates a bacterial strain’s capacity to lyse host red blood cells (RBCs), resulting in the release of intracellular components such as hemoglobin. This activity is primarily mediated by hemolysins, which disrupt host cell membranes and lead to erythrocyte lysis. Hemolytic activity is typically assessed using blood agar media, where the outcomes are categorized into three distinct phenotypes: γ-hemolysis (no lysis) is considered acceptable, β-hemolysis (complete lysis) is unacceptable, and α-hemolysis (partial lysis) is conditionally acceptable depending on the absence of associated virulence genes [44, 45].

### Safety Assessment of Metabolic Activities (D-lactate, Biogenic amine, Bile salt hydrolase activity)

The assessment of metabolic safety focuses on identifying metabolites produced by the microorganism that may exert adverse effects on the host. Certain jurisdictions compared in this review, including the US FDA, HC, Thai FDA, and ANVISA, require the safety assessment of metabolic activities for NMs intended as food ingredients (Table 3). Particular attention is given to the production of harmful metabolites, such as biogenic amines and D-lactate, which have been associated with toxicological and physiological risks [46-50]. In addition, specific enzymatic activities, including bile salt hydrolase (BSH), are evaluated due to their potential impact on host metabolic pathways, particularly bile acid metabolism [51, 52]. While metabolites like D-lactate and biogenic amines, as well as BSH activity, are generally benign to healthy individuals under typical consumption conditions, caution may be warranted for susceptible populations, such as neonates or immunocompromised patients, particularly under elevated exposure [47, 53, 54]. Overall, these evaluations ensure that the metabolic profile of the microorganism does not produce compounds that could compromise human health.

### Toxicological Evaluation via *In Vitro* and *In Vivo* Assessments

Toxicological data provides experimental evidence to assess potential adverse effects through genotoxicity and subchronic toxicity studies. Therefore, most jurisdictions compared in this review require the toxicological evaluation of NMs intended as food ingredients (Table 3). This evaluation is a critical component of the safety assessment and is commonly conducted using both *in vitro* and *in vivo* approaches in accordance with internationally recognized guidelines, such as the Organization for Economic Co-operation and Development (OECD) Test Guidelines. Toxicological evaluation generally follows a tiered approach, starting with core studies such as acute toxicity tests (*e.g.*, OECD TG 420, 423, 425), repeated-dose toxicity tests (*e.g.*, OECD TG 408), and genotoxicity assays (*e.g.*, Ames test, chromosomal aberration tests, and micronucleus assays) [19, 55-58]. According to EFSA, genotoxicity testing for microorganisms may be required depending on taxonomic and hazard characteristics. This is typically performed using both cell-free supernatants and cell lysates, with confirmation of efficient cell lysis. However, for microorganisms with a well-documented history of safe use, certain toxicological studies may be reduced or waived, depending on the weight of evidence and existing knowledge.

### Safety Assessment of Non-Viable Bacteria and Microbial Metabolites as Novel Food Ingredients

The safety assessment criteria, along with relevant examples of non-viable bacteria and microbial metabolites as novel food ingredients, were reviewed by investigating the regulations and guidelines across eight major jurisdictions including the United States (Food and Drug Administration, FDA), the European Union (European Food Safety Authority, EFSA), Canada (Health Canada, HC), Australia/New Zealand (Food Standards Australia New Zealand, FSANZ), Japan (The Food Safety Commission of Japan, FSCJ & Consumer Affairs Agency, CAA), Thailand (Thailand Food and Drug Administration, Thai FDA), India (Food Safety and Standards Authority of India, FSSAI), and Brazil (Agência Nacional de Vigilância Sanitária, ANVISA).

### Safety Assessment Criteria

As summarized in Table 4, non-viable bacteria and microbial metabolites lack a dedicated “postbiotic” legal category in most jurisdictions; instead, they undergo a case-by-case evaluation within novel food, food additive, or functional food frameworks. Across jurisdictions, safety assessment generally requires the following core datasets: (1) unambiguous taxonomic identification at the species and strain levels, increasingly supported by WGS; (2) characterization of genes of potential concern, including acquired antimicrobial resistance genes, toxin-encoding genes, and virulence factors; (3) for non-viable bacteria, verification of non-viability through validated methods (*e.g.*, plate counts, viability staining) with defined limits of detection (*e.g.*, <10 CFU/g); (4) a comprehensive description of the manufacturing process, including raw materials, fermentation conditions, inactivation methods (heat, pressure, or chemical treatment) with process validation, and purification/formulation steps; (5) detailed specifications covering identity, microbial purity, chemical composition, moisture, and pH, and for metabolites, levels of process-related impurities such as residual proteins, endotoxins, and solvents; (6) a toxicological evaluation, typically consisting of a 90-day repeated-dose oral toxicity study in rodents with appropriate dose selection and comprehensive endpoint assessments, supplemented by a genotoxicity battery; and (7) a dietary exposure assessment based on proposed food uses and target populations [19, 55-59].

### Representative Precedents of Non-viable Bacteria

For non-viable bacteria, the US established a strong precedent with heat-killed *Clostridium tyrobutyricum* ASM#19 (GRAS GRN No. 1129), which received an FDA “no questions” letter in 2024 for use as a protein source in foods and beverages at inclusion levels up to 90%. The FDA's response validated the approach of leveraging WGS for taxonomic confirmation, process validation for non-viability, and 90-day toxicity studies demonstrating no adverse effects [57]. Concurrently, the EU authorized pasteurized *A. muciniphila* as a novel food in 2021, with EFSA concluding safety at daily intakes up to 3.4 × 10^10^ cells (derived from a 90-day rat NOAEL with a 200-fold uncertainty factor), provided that viable cells remain below 10 CFU/g (the limit of detection). This precedent integrated WGS, AMR gene evaluation, extensive toxicology, and human intervention data, setting a rigorous standard for inactivated bacterial biomasses. Canada approved Nutractis™, a protein matrix containing heat-killed debris of *Lactobacillus kefiranofaciens* strain R2C2, for use in butter, cheese spreads and whipped cream, demonstrating acceptance of inactivated cells as functional food ingredients [60]. Japan leads in commercialization via the Foods with Function Claims (FFC) system, with heat-killed *L. plantarum* L-137 (HK L-137) supported by human safety trials up to 12 months showing no adverse events [61]. Furthermore, Thailand's approval of LAC-Shield (heat-killed *Lacticaseibacillus paracasei* MCC1849) and India's clearance of LC-Plasma (heat-killed *Lactococcus lactis* strain Plasma) demonstrate growing regulatory acceptance of heat-inactivated bacterial preparations as standard food ingredients.

### Representative Precedents of Microbial Metabolites

For microbial metabolites, fermentation-derived human milk oligosaccharides (HMOs), particularly 2'-fucosyllactose (2'-FL), exemplify regulatory harmonization. Multiple jurisdictions have authorized HMOs derived from various genetically modified *Escherichia coli* strains for infant formula, applying consistent safety principles: production strain characterization (genetic modifications, stability, and absence of pathogenicity), process validation ensuring the removal or inactivation of production strains, impurity analysis, comprehensive toxicology including developmental studies, and allergenicity assessment [62-65]. Especially, the assessment of allergenicity is performed to determine whether a novel microorganism-derived food ingredient has the potential to induce allergic reactions in susceptible individuals [19, 56]. Rather than relying on a single experimental assay, allergenicity is generally evaluated using a weight-of-evidence approach. The assessment typically considers the identity and history of safe use of the production microorganism, characterization of residual proteins in the final product, amino acid sequence homology to known allergens using established allergen databases such as AllergenOnline, anticipated dietary exposure, and available human or published evidence [39].

Concurrently, Brazil's ANVISA adopted Resolution RDC No. 839 in December 2023 (entering into force in March 2024), establishing a modernized framework that explicitly recognizes microorganisms and their “derived components” as potential novel foods or ingredients [58].

Overall, the regulatory landscape for non-viable bacteria and metabolites is thus evolving toward greater harmonization in safety assessment principles. The precedents established by heat-killed *C. tyrobutyricum* (USA), pasteurised *A. muciniphila* (EU), Nutractis™ (Canada), HK L-137 (Japan), LAC-Shield (Thailand) and LC-Plasma (India) collectively demonstrate regulatory acceptance of non-viable bacteria as safe food ingredients when supported by robust strain-level characterization and toxicological data. Simultaneously, the widespread authorization of microbial metabolites like 2’-FL and other HMOs across multiple markets illustrates a convergent approach to the safety evaluation of microbial metabolites, centered on production strain safety, product purity and comprehensive toxicology.

## Conclusion

Despite the expanded utilization of novel microorganism-derived ingredients in food, there is currently no globally harmonized protocol for their safety assessment, and significant international discrepancies persist regarding the required scope of empirical data and the rigor of risk assessment methodologies. Continued efforts to harmonize regulatory terminology, safety evaluation criteria, and risk assessment methodologies will be essential to support the safe and sustainable development of these novel ingredients.

**Fig. 2:**
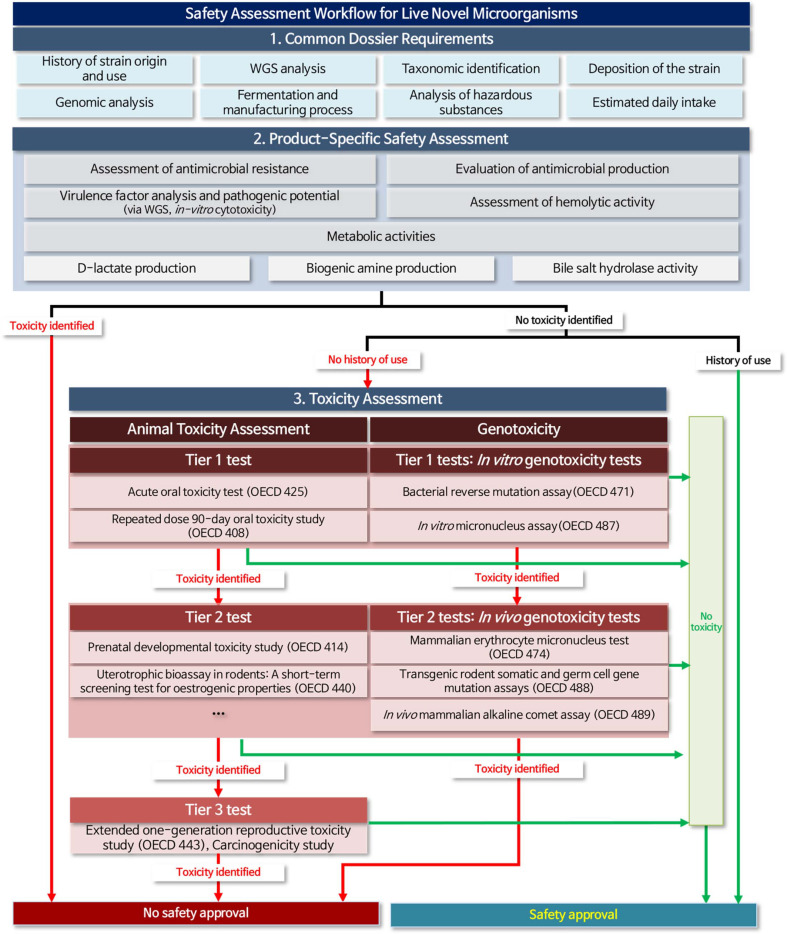
Proposed safety assessment workflow for live novel microorganisms as food ingredients. Following a product-specific safety assessment, strains with toxicity concerns are excluded from food use. Conversely, strains with no identified toxicity proceed to a further safety evaluation based on their history of use. Strains with a documented history of use receive immediate safety approval, whereas those without a history of use undergo a tiered toxicity assessment. Tier 1 tests include parallel acute oral and repeated dose 90-day oral toxicity tests alongside *in vitro* genotoxicity assays; Tier 2 tests add prenatal developmental/uterotrophic bioassay in rodents toxicity and *in vivo* genotoxicity tests; Tier 3 tests include extended one-generation reproductive toxicity and carcinogenicity studies. At any given tier, the absence of toxicity leads to safety approval, while toxicity that persists through Tier 3 results in the denial of safety approval.

**Fig. 3:**
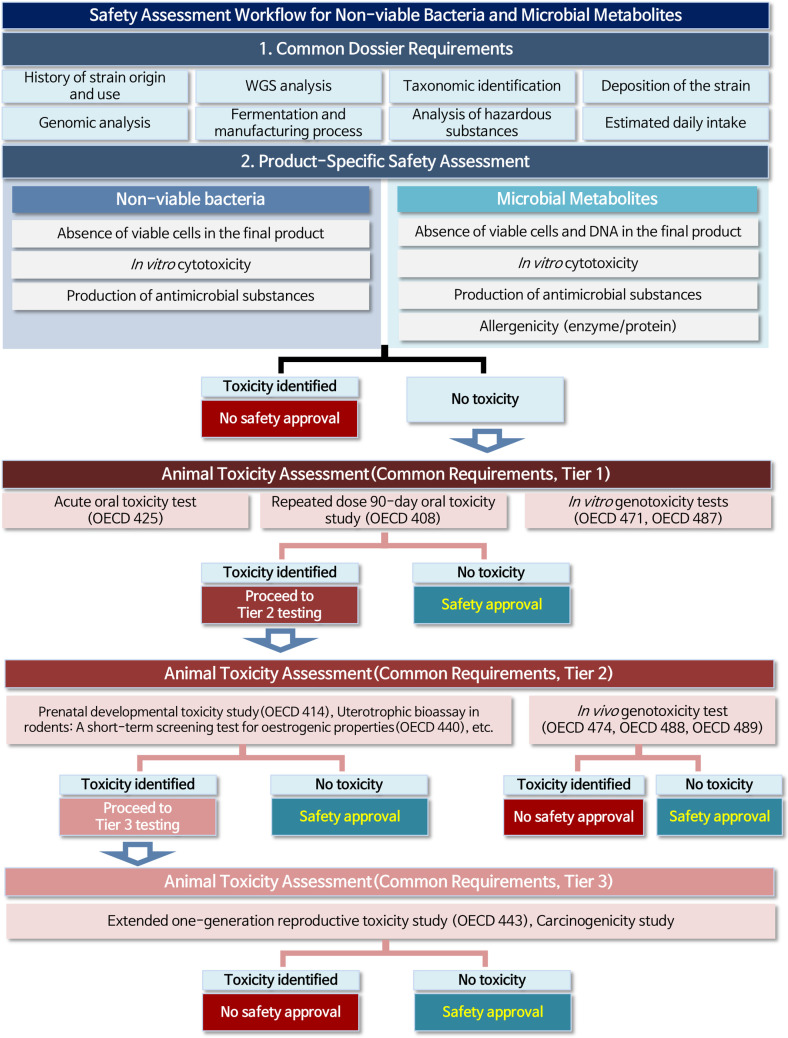
Proposed safety assessment workflow for non-viable bacteria and microbial metabolites as food ingredients. Following a product-specific safety assessment, ingredients presenting safety concerns are considered unsuitable for food use. For ingredients without identified safety concerns, the proposed workflow applies a tiered toxicity assessment for non-viable bacteria and microbial metabolites. Tier 1 tests comprise acute oral toxicity, repeated dose 90-day oral toxicity study, and *in vitro* genotoxicity tests, which are followed by Tier 2 and Tier 3 evaluations when additional toxicological assessment is warranted. The final safety decision is based on the cumulative evidence generated throughout the tiered testing process.

Therefore, we propose a safety assessment workflow for live NMs (Fig. 2), non-viable bacteria (postbiotics), and purified metabolites as food ingredients (Fig. 3) based on a comprehensive review of international regulatory frameworks. The proposed framework integrates common dossier requirements, product-specific safety assessment, and a tiered toxicity testing strategy based on OECD guidelines. By incorporating essential assessments such as *in vitro* cytotoxicity, antimicrobial activity, and tiered toxicological testing, this workflow provides a scientifically grounded and harmonized approach for evaluating the safety of novel microbial-derived food ingredients.

## Figures and Tables

**Table 1:** Definition and classification of microorganism-derived food ingredients.

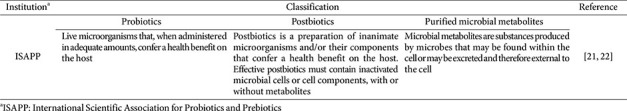

**Table 2:** Overview of regulatory authorities in the selected jurisdictions.

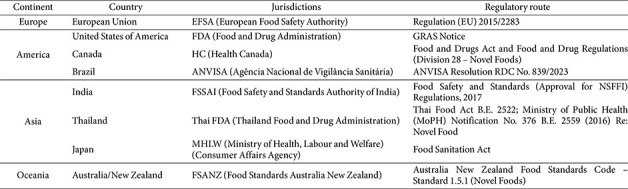

**Table 3:** Comparison of safety assessment requirements for live novel microorganisms across regulatory systems.

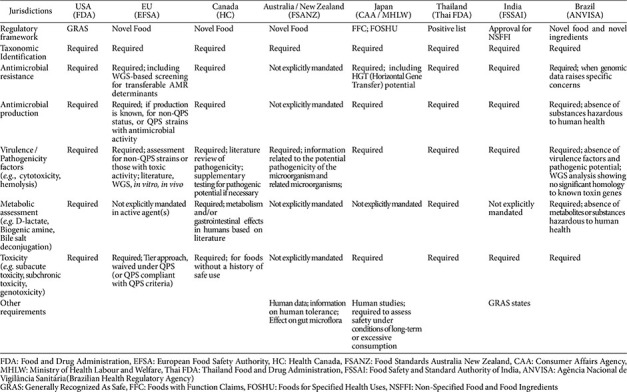

**Table 4:** Representative precedents of the non-viable bacteria and microbial metabolites as food ingredients in eight jurisdictions.